# A Comparison of Eye Movement Measures across Reading Efficiency Quartile Groups in Elementary, Middle, and High School Students in the U.S.

**DOI:** 10.16910/jemr.10.4.5

**Published:** 2017-12-02

**Authors:** Alexandra N. Spichtig, Jeffrey P. Pascoe, John D. Ferrara, Christian Vorstius

**Affiliations:** Reading Plus,Winooski, Vermont, United States of America; Bergische Universität Wuppertal, Germany

**Keywords:** eye movement, reading, silent reading efficiency, fluency, automaticity, children, saccades, individual differences

## Abstract

This cross-sectional study examined eye movements during reading across grades in stu-dents with differing levels of reading efficiency. Eye-movement recordings were obtained while students in grades 2, 4, 6, 8, 10, and 12 silently read normed grade-leveled texts with demonstrated comprehension. Recordings from students in each reading rate quartile at each grade level were compared to characterize differences in reading rate, number of fixations, number of regressions, and fixation durations. Comparisons indicated that stu-dents in higher reading rate quartiles made fewer fixations and regressions per word, and had shorter fixation durations. These indices of greater efficiency were also characteristic of students in upper as compared to lower grades, with two exceptions: (a) between grades 6 and 8, fixations and regressions increased while reading rates stagnated and fixation durations continued to decline, and (b) beyond grade 6 there was relatively little growth in the reading efficiency of students in the lower two reading rate quartiles. These results suggest that declines in fixation duration across grades may in part reflect broader matura-tional processes, while higher fixation and regression rates may distinguish students who continue to struggle with word recognition during their high school years.

## Introduction

The process of reading involves a succession of eye movements (saccades) that strategically position the eyes at successive points along lines of print, alternating with fixations (times of relative stability of the eyes) during which visual information is captured. The number of fixations per word, fixation durations, number of regressive saccades (right-to-left in English), and the amount of textual information perceived with each fixation (perceptual span), are some common reading-related eye movement measures with values that typically shift with age and in relation to reading proficiency. Many features of eye movements during reading change as reading skills increase over time. More experienced readers generally read text more quickly, make fewer fixations and regressions per word, have shorter fixation durations, utilize a wider perceptual span, and make longer saccades [ 1, 2, 3, 4, 5, 6, 7, 8 ]. These wellestablished patterns of development have been characterized in various ways, such as across school grades [ 6, 9 ], in relation to oral versus silent reading [ 8, 10, 11, 12 ], across writing systems [ 13 ], in older readers [ 14, 15 ], and in readers who have become more efficient as a result of structured silent reading practice [ 16, 17]. Importantly, a number of these studies have used connected text and included comprehension measures to ensure that eye movement recordings were obtained during authentic, productive reading.

Other features of eye movement behavior during reading seem to become fairly well established in the early stages of reading development, and thus may be more closely related to early-developing sensorimotor, perceptual, and attentional mechanisms rather than capacities with a more protracted developmental time course (e.g., [ 18 ]). Using, for example, a disappearing text paradigm in which words vanish shortly (e.g., 60 ms) after they are fixated, it was found that children seem to be as capable as adults in terms of the speed with which they can extract visual information from text during a single fixation [ 19 ]. Concerning the location at which the eyes first land within a word, the initial fixations of beginning readers tend to land near to the start of a word [ 20 ]. This is an efficient strategy given the beginning reader’s tendency to refixate most words during lexical identification. Beyond this initial stage, however, the location at which the eyes first land within a word tends to be similar across a range of word lengths in both young readers and adults [ 21 ]. This in turn suggests that saccadic targeting and the use of parafoveal vision to guide saccadic targeting during reading are capabilities that become established to a considerable degree in the early stages of reading development. Also during these early stages, the perceptual span enlarges and becomes asymmetrical; extending further to the right of each fixation in languages that present text from left to right [ 2, 22 ]. Evidence that this reflects an attentional process includes the observations that the properties of words in the parafoveal region can influence fixation durations [ 23 ], perceptual span narrows when reading more difficult text [ 22 ], and the direction of perceptual asymmetry alternates as appropriate in bilinguals presented with text in languages that read from left to right versus right to left [ 24 ].

Less well studied are developmental changes in eye movements during reading in school-age peers with differing levels of reading efficiency. This is of interest because, as the foregoing suggests, age-related changes in eye movements during reading are very likely a consequence of both maturational processes (e.g., increasing sensorimotor control and cognitive capacity) and accumulating reading experience (e.g., [ 1, 25 ]). The manner in which maturation and reading experience combine in more versus less efficient readers, however, is not well understood. Disentangling the contributions of these two factors is challenging, but useful insights might be gained by characterizing the reading related eye movements of students at different grade levels, and then comparing these measures across groups of students within and across grades who demonstrate different levels of reading efficiency. The present research was undertaken for this purpose; i.e., to describe and explore related parameters of differently efficient readers at different points in reading development.

Eye movements were recorded while students silently read grade-leveled texts and then answered comprehension questions. Only recordings with adequate comprehension were included in the analyses since the purpose of this study was to evaluate differences in reading efficiency measures during authentic, productive reading. Included were students at six different grade levels ranging from grade 2 to grade 12. In an earlier report [ 6 ], grade level means for reading rate and the three eye movement measures (fixations, regressions, and fixation duration) were described in these populations and compared to data reported in 1960. For the present report, students in each grade were divided into four reading rate quartile groups representing four different levels of reading efficiency, and data were analyzed using quartile membership as a factor. Reading rate was used to establish efficiency quartile groups with the idea that fixation duration, in combination with fixation and regression counts are the constituents of reading rate. This enabled consideration of the following questions: (a) How do reading rate and eye movement measures during reading differ across students who have reached the same grade but exhibit different levels of reading efficiency? (b) How do the developmental trajectories of these measures differ across grades in students with different levels of reading efficiency?

## Methods

### Participants

Eye-movement recordings from 2,203 students in grades 2, 4, 6, 8, 10, and 12 were collected in the spring of 2011. The study included participants from 34 schools in 16 states representing all geographic regions of the U.S. Participating schools were asked to select a representative sample of students comprising those who had scored below-average, average, and above-average on the reading/language arts assessment used in their state (many states develop their own assessment to monitor reading comprehension in schools state-wide). Assessment data were obtained from 93% of the schools and showed that 69.7% of the participating students had attained proficiency on their assessment. There was an approximately equal distribution of males and females in each grade. Data from students who were classified either as English Learners or eligible for special education services were not included in the analyses. Satisfactory recordings were obtained from 91% of the participants, comprising between 223 and 479 students at each grade level. The racial and ethnic distribution of the sample (White, 60%; Black, 16%; Hispanic, 20%; Asian, 3%; and other, 1%) approximated the national distribution when the data were collected [ 26 ]. The percentage of students eligible for free/reduced price lunch (49%) was nearly identical to the national average [ 27 ]. Additional details were described in another article based on the same data set [ 6 ].

### Procedure

Reading-related eye movement data were captured using a portable eye movement recording system (Visagraph) [ 28 ]. This relatively simple system uses goggles fitted with infrared emitters and sensors to measure binocular eye movements (corneal reflections) at a sampling rate of 60 Hz. Despite its simplicity, the Visagraph yields reading related eye-movement data comparable to more sophisticated eye movement recording systems with regard to the general measures reported in this article [ 29 ]. For quantifying eye-movement behavior at the group level, the eye-movement data captured by the Visagraph is reliable when following standardized procedures and given an adequate sample size, as was the case in the current research [ 30 ].

Recordings were collected while students read five normed grade level passages (one practice trial at a level that was two grades below a student’s grade level, followed by four test trials at the student’s grade level). Students were instructed to read silently, and reminded of this if they started reading aloud during the practice trial. The passages were either 50-words in length with a 16-point font size (grade 2), or 100 words in length with a 14-point font size (grades 4, 6, 8, 10, and 12), and were presented using a full-justified Times New Roman typeface. All passages were developed using an assortment of age-appropriate readability formulas and had been used previously in cross-sectional readingrelated eye-movement research (see Spichtig et. al., 2016 and Taylor, 1965 for more details regarding the test passages). The grade levels of the passages were also evaluated using the Lexile Framework [ 31 ], and an analysis of word frequency was performed for each of the test passages using the SUBTLEXUS corpus [ [Bibr b32]].

Performance data were calculated automatically by the Visagraph software, yielding estimates of (a) silent reading rate (expressed in words per minute; wpm), (b) number of fixations, (c) number of regressions, and (d) average fixation duration (measured in milliseconds; ms). Fixation and regression measures were derived for each individual by dividing the fixation and regression counts by the number of words in each passage and then averaging these values across passages. Therefore, the presented values represent the mean fixation and regression counts per word. Due to limitations of the recording system, the reported fixation durations include saccade time (~20–40 ms), and only short-range regressions (up to about three words in length) were included in the regression count.

To ensure that reading performances were genuine, a comprehension check followed each passage. Students were asked to answer 10 true/false comprehension questions that were developed for use with the grade level passages [ 9 ]. During initial testing of the comprehension items, it was found that students who had not read a passage and answered by guessing averaged 56% correct, while those who had first read the passage averaged 88% correct. On the basis of these results, 70% correct was selected as the criterion for adequate comprehension, and eye-movement recordings were only regarded as valid if a student achieved or exceeded this criterion. In other words, all reading rate and eye movement measures reported here are based on silent reading performances on passages where adequate comprehension was demonstrated.

### Data Analysis

For each student, performance data from all valid test passages (i.e., passages with demonstrated comprehension) were averaged into a single mean score for each measure. These mean scores were then used in the analyses. The mean reading rate scores were also used to divide students into the four reading rate quartile groups.

Differences in each reading efficiency measure (silent reading rate, fixation count, regression count, and fixation duration) across grades and reading rate quartile groups were evaluated utilizing linear models fitted using generalized least squares. Within the R environment for statistical computing [ 33], the *gls* function was used in combination with the *varIdent* function from the *nlme* package [ 34]. Grade and reading rate quartile were specified as fixed factors, and successive difference contrasts [ 35 ] were used to evaluate differences in reading efficiency measures from grade to grade and between quartiles as well as interactions between these factors. The *varIdent* function allows different variances, one for each level of a factor, safeguarding against violations of homogeneity of variance. All of the comparisons were a priori, orthogonal, and within the allowable degrees of freedom offered by the design. The inferential statistics reported are the actual results from the analyses. Because multiple comparisons were made, the Benjamini-Hochberg procedure was used to control for the false discovery rate [ 36 ]. Comparison contrasts were rank ordered by *p*-values and compared to (*i/m*)*Q*, where *i* = rank, *m* = number of comparisons, and *Q* = 0.05 (false discovery rate).

## Results

Ninety-one percent (*n* = 2,009) of the participants in this study completed at least one and as many as four valid recordings; i.e., one or more recordings that were interpretable and met or exceeded the 70% criterion on the comprehension probe that followed. Students met these criteria on one (19.6%), two (26%), three (25.1%) or four (20.5%) of their test trials. On average, participants completed 2.3 valid recordings, with some variation across grades (grade 2, 2.5; grade 4, 1.8; grade 6, 2.4; grade 8, 2.3; grade 10, 2.3; grade 12, 2.5). The Lexile scores, mean word lengths, and average word frequencies of the passages used at each grade level are shown in Table 1.The SUBTLEXUS corpus contained 98.3% of the words in the passages. The Lexile scores, mean word lengths, and average word frequencies of the passages used at each grade level are shown in Table 1. The SUBTLEXUS corpus contained 98.3% of the words in the passages.

**Table 1:** Lexile Scores, Mean Word Lengths, and Word Frequencies of Passages

Grade		Lexile Score		Word Length				MLWF		SBTLWF
				*M*		*SD*		*M*	*SD*	All		Unique
2		473		4.13		1.69		3.67	0.18	5605.16		3947.95
4		780		4.37		2.00		3.65	0.05	6286.11		2959.98
6		930		4.72		2.05		3.34	0.11	5126.12		3059.03
8		1086		4.86		2.30		3.16	0.11	4263.02		2393.99
10		1206		4.96		2.62		3.32	0.04	4561.50		2746.66
12		1243		5.35		2.90		3.25	0.08	4576.12		2496.02

Notes: MLWF is the mean of the log word frequencies based on the Lexile corpus (Stenner et al., 2007). SBTLWF is the word frequen-cy per million words based on the SUBTLEXUS corpus (Brysbaert und New, 2009). Shown are the averages of all the words in a passage, and of all the unique words in a passage.

The results of the linear model analyses for each measure are described in the following sections. Note that in each case, only orthogonal comparisons were made; i.e., between adjacent grades and quartiles. The statistics shown in the tables are the actual output of the linear model analyses. The *p*-values reflect the probability that a given difference estimate is significantly different from zero.

**Figure 1:**
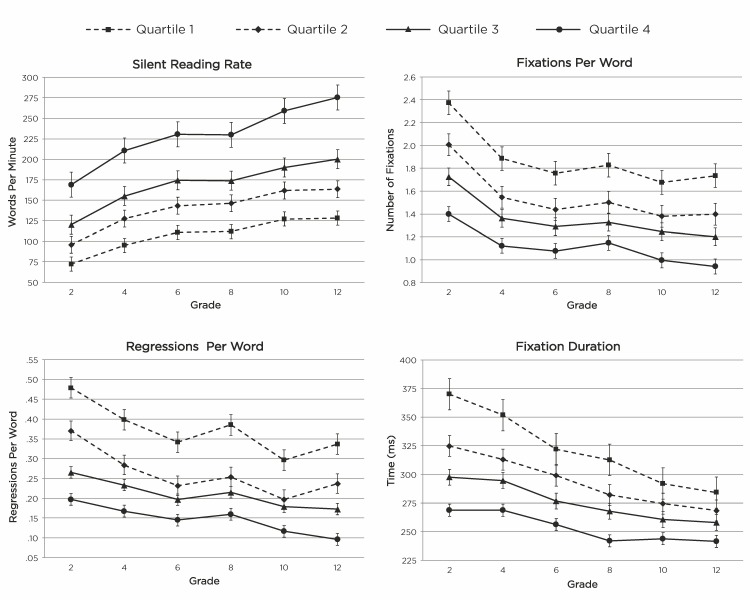
Reading Efficiency Measures Across Grades and Reading Rate Quartiles.

Shown in Figure 1 are the values for each measure at each grade level in each of the four reading rate quartiles. The actual means, standard deviations, and 95% confidence intervals at each data point are presented in Table 2. The reported values for fixation duration include saccade time (~20-40 ms). Results of the linear model analyses comparing estimated differences in each measure across adjacent grades, adjacent quartiles, and interactions between these factors, are shown in Table 3(reading rate), Table 4(fixations per word), Table 5(regressions per word), Table 6(fixation durations).

**Table 2:** Silent Reading Efficiency Measures Across Grades and Quartiles

	Quartile 1			Quartile 2			Quartile 3			Quartile 4
Grade (*n*)	Mean	SD	95% CI	Mean	SD	95% CI	Mean	SD	95% CI	Mean	SD	95% CI
Reading Rate (wpm)
2 (n=379)	72	11	70, 75	96	6	95, 97	120	8	119, 122	169	32	162, 175
4 (n=383)	95	12	92, 97	128	8	126, 129	155	8	154, 157	211	45	202,220
6 (n=294)	111	15	107, 114	143	9	141, 145	174	9	172, 176	230	44	220, 241
8 (n=479)	112	18	109, 125	146	7	145, 148	174	10	172, 176	230	38	223, 237
10 (n=251)	127	16	123, 131	162	8	160, 164	190	11	187, 193	259	45	248, 270
12 (n=223)	128	20	123, 134	163	5	162, 165	200	13	197, 204	275	36	166, 285
Fixations Per Word
2 (n=379)	2.37	0.37	[2.30, 2.45]	2.01	0.32	[1.94, 2.07]	1.73	0.19	[1.69, 1.76]	1.40	0.22	[1.35, 1.44]
4 (n=383)	1.88	0.37	[1.81, 1.96]	1.55	0.18	[1.51, 1.58]	1.36	0.20	[1.32, 1.40]	1.12	0.20	[1.08, 1.16]
6 (n=294)	1.76	0.34	[1.68, 1.84]	1.44	0.19	[1.40, 1.48]	1.29	0.17	[1.25, 1.33]	1.08	0.19	[1.03, 1.12]
8 (n=479)	1.83	0.40	[1.76, 1.90]	1.50	0.26	[1.46, 1.55]	1.33	0.19	[1.29, 1.36]	1.15	0.25	[1.10, 1.19]
10 (n=251)	1.68	0.28	[1.60, 1.75]	1.38	0.16	[1.34, 1.42]	1.24	0.14	[1.21, 1.28]	0.99	0.17	[0.95, 1.04]
12 (n=223)	1.74	0.32	[1.65, 1.82]	1.40	0.17	[1.35, 1.44]	1.20	0.17	[1.16, 1.24]	0.94	0.14	[0.90, 0.98]
Regressions Per Word
2 (n=379)	0.48	0.19	[0.44, 0.52]	0.37	0.18	[0.33, 0.41]	0.27	0.11	[0.24, 0.29]	0.20	0.09	[0.18, 0.22]
4 (n=383)	0.40	0.18	[0.36, 0.44]	0.28	0.10	[0.26, 0.30]	0.23	0.11	[0.21, 0.26]	0.17	0.08	[0.15, 0.18]
6 (n=294)	0.34	0.15	[0.31, 0.38]	0.23	0.09	[0.21, 0.25]	0.20	0.08	[0.18, 0.21]	0.14	0.07	[0.13, 0.16]
8 (n=479)	0.38	0.20	[0.35, 0.42]	0.25	0.12	[0.23, 0.28]	0.22	0.09	[0.20, 0.23]	0.16	0.11	[0.14, 0.18]
10 (n=251)	0.30	0.11	[0.27, 0.32]	0.20	0.09	[0.17, 0.22]	0.18	0.08	[0.16, 0.20]	0.12	0.06	[0.10, 0.13]
12 (n=223)	0.34	0.16	[0.29, 0.38]	0.24	0.10	[0.21, 0.26]	0.17	0.08	[0.15, 0.20]	0.10	0.06	[0.08, 0.11]
Fixation Durations (ms)
2 (n=379)	370	73	355, 285	325	46	315, 334	298	31	291, 304	269	35	262, 267
4 (n=383)	352	49	342, 362	313	40	305, 321	295	52	284, 305	269	37	261,276
6 (n=294)	322	39	313, 331	299	38	290, 308	277	33	269, 284	256	35	248, 264
8 (n=479)	313	68	300, 325	282	39	275, 289	268	31	262, 274	242	36	235, 249
10 (n=251)	292	34	284, 301	274	27	268, 281	261	27	254, 267	244	25	238, 250
12 (n=223)	284	42	273, 295	269	32	260, 277	258	31	250, 266	242	28	234, 249

**Table 3:** Differences in Reading Rate Between Grades and Reading Rate Quartiles

	Difference in reading rate
	Estimate	SE	*t*-value	*p*
Intercept	161.1	0.52	309.9	< .001
Grade comparisons
4th vs. 2nd	32.5	1.52	21.4	< .001
6th vs. 4th	17.7	1.86	9.5	< .001
8th vs. 6th	0.6	1.73	0.3	*ns*
10th vs. 8th	19.4	1.85	10.5	< .001
12th vs. 10th	7.3	2.13	3.4	< .001
Quartile comparisons
Q2 vs. Q1	32.3	0.81	39.7	< .001
Q3 vs. Q2	29.4	0.58	55.6	< .001
Q4 vs. Q3	60.0	1.92	31.2	< .001
Grade x Quartile interactions
4th vs. 2nd by Q2-Q1	9.1	1.96	4.6	< .001
6th vs. 4th by Q2-Q1	-0.2	2.52	-0.1	*ns*
8th vs. 6th by Q2-Q1	2.1	2.71	0.8	*ns*
10th vs. 8th by Q2-Q1	-0.2	2.88	-0.1	*ns*
12th vs. 10th by Q2-Q1	0.7	3.56	0.2	*ns*
4th vs. 2nd by Q3-Q2	2.5	1.55	1.6	*ns*
6th vs. 4th by Q3-Q2	3.8	1.86	2.1	0.039
8th vs. 6th by Q3-Q2	-3.3	1.83	-1.8	*ns*
10th vs. 8th by Q3-Q2	0.3	2.04	0.1	*ns*
12th vs. 10th by Q3-Q2	9.0	2.53	3.6	< .001
4th vs. 2nd by Q4-Q3	6.9	5.74	1.2	*ns*
6th vs. 4th by Q4-Q3	0.8	7.01	0.1	*ns*
8th vs. 6th by Q4-Q3	-0.4	6.38	-0.1	*ns*
10th vs. 8th by Q4-Q3	13.4	6.84	2.0	0.049
12th vs. 10th by Q4-Q3	6.1	7.72	0.8	*ns*

**Table 4:** Differences in Fixations Per Word Between Grades and Reading Rate Quartiles

	Difference in fixations per word
	Estimate	SE	*t*-value	*p*
Intercept	1.47	0.005	269	< .001
Grade comparisons
4th vs. 2nd	-0.39	0.019	-20.4	< .001
6th vs. 4th	-0.09	0.019	-4.7	< .001
8th vs. 6th	0.06	0.019	3.3	0.001
10th vs. 8th	-0.13	0.018	-7.3	< .001
12th vs. 10th	0	0.019	-0.3	*ns*
Quartile comparisons
Q2 vs. Q1	-0.33	0.018	-18.1	< .001
Q3 vs. Q2	-0.19	0.012	-15	< .001
Q4 vs. Q3	-0.24	0.012	-20.4	< .001
Grade x Quartile interactions
4th vs. 2nd by Q2-Q1	0.04	0.064	0.5	*ns*
6th vs. 4th by Q2-Q1	0.04	0.062	0.7	*ns*
8th vs. 6th by Q2-Q1	-0.02	0.063	2.2	*ns*
10th vs. 8th by Q2-Q1	0.03	0.059	0.6	*ns*
12th vs. 10th by Q2-Q1	-0.05	0.063	-0.7	*ns*
4th vs. 2nd by Q3-Q2	0.1	0.046	2.2	0.029
6th vs. 4th by Q3-Q2	0.02	0.04	0.5	*ns*
8th vs. 6th by Q3-Q2	-0.02	0.041	-0.7	*ns*
10th vs. 8th by Q3-Q2	0.04	0.039	1	*ns*
12th vs. 10th by Q3-Q2	-0.06	0.041	-1.3	*ns*
4th vs. 2nd by Q4-Q3	0.08	0.042	1.9	*ns*
6th vs. 4th by Q4-Q3	0.02	0.042	0.6	*ns*
8th vs. 6th by Q4-Q3	0.04	0.041	0.9	*ns*
10th vs. 8th by Q4-Q3	-0.07	0.039	-1.7	*ns*
12th vs. 10th by Q4-Q3	-0.01	0.04	-0.3	*ns*

**Table 5:** Differences in Number of Regressions Per Word Between Grades and Reading Rate Quartiles

	Difference in regressions per word
	Estimate	SE	*t*-value	*p*
Intercept	0.25	0.003	93.8	< .001
Grade comparisons
4th vs. 2nd	-0.06	0.010	-5.6	< .001
6th vs. 4th	-0.04	0.009	-4.9	< .001
8th vs. 6th	0.03	0.009	3	0.003
10th vs. 8th	-0.06	0.008	-7	< .001
12th vs. 10th	0.01	0.009	1.5	*ns*
Quartile comparisons
Q2 vs. Q1	-0.11	0.009	-12.1	< .001
Q3 vs. Q2	-0.05	0.007	-8.1	< .001
Q4 vs. Q3	-0.06	0.005	-11.3	< .001
Grade x Quartile interactions
4th vs. 2nd by Q2-Q1	-0.01	0.034	-0.2	*ns*
6th vs. 4th by Q2-Q1	0.01	0.029	0.3	*ns*
8th vs. 6th by Q2-Q1	-0.02	0.029	-0.8	*ns*
10th vs. 8th by Q2-Q1	0.04	0.027	1.3	*ns*
12th vs. 10th by Q2-Q1	-0.01	0.031	-0.2	*ns*
4th vs. 2nd by Q3-Q2	0.06	0.026	2.4	0.015
6th vs. 4th by Q3-Q2	0.01	0.020	0.7	*ns*
8th vs. 6th by Q3-Q2	-0.01	0.019	-0.4	*ns*
10th vs. 8th by Q3-Q2	0.01	0.02	0.7	*ns*
12th vs. 10th by Q3-Q2	-0.04	0.023	-1.7	*ns*
4th vs. 2nd by Q4-Q3	-0.01	0.020	-0.3	*ns*
6th vs. 4th by Q4-Q3	0.01	0.018	0.7	*ns*
8th vs. 6th by Q4-Q3	0	0.018	0.1	*ns*
10th vs. 8th by Q4-Q3	0	0.018	-0.1	*ns*
12th vs. 10th by Q4-Q3	-0.02	0.018	-1.1	*ns*

**Table 6:** Differences in Fixation Duration Between Grades and Reading Rate Quartiles

	Difference in fixation duration
	Estimate	SE	*t*-value	*p*
Intercept	286.1	0.89	320.5	< .001
Grade comparisons
4th vs. 2nd	-7.9	3.42	-2.3	0.022
6th vs. 4th	-18.5	3.11	-5.9	< .001
8th vs. 6th	-12.2	2.97	-4.1	< .001
10th vs. 8th	-8.5	2.77	-3.1	0.002
12th vs. 10th	-4.7	2.86	-1.6	*ns*
Quartile comparisons
Q2 vs. Q1	-28.5	2.83	-10.1	< .001
Q3 vs. Q2	-17.5	2.29	-7.6	< .001
Q4 vs. Q3	-22.4	2.17	-10.3	< .001
Grade x Quartile interactions
4th vs. 2nd by Q2-Q1	6.9	10.98	0.6	*ns*
6th vs. 4th by Q2-Q1	10	8.96	1.1	*ns*
8th vs. 6th by Q2-Q1	-4.9	9.51	-0.5	*ns*
10th vs. 8th by Q2-Q1	13	9.03	1.4	*ns*
12th vs. 10th by Q2-Q1	2.5	8.85	0.3	*ns*
4th vs. 2nd by Q3-Q2	8.3	8.84	0.9	*ns*
6th vs. 4th by Q3-Q2	-2.0	8.75	-0.2	*ns*
8th vs. 6th by Q3-Q2	7.7	7.20	1.1	*ns*
10th vs. 8th by Q3-Q2	1.3	6.70	0.2	*ns*
12th vs. 10th by Q3-Q2	2.0	7.71	0.3	*ns*
4th vs. 2nd by Q4-Q3	3.2	8.17	0.4	*ns*
6th vs. 4th by Q4-Q3	6.3	8.65	0.7	*ns*
8th vs. 6th by Q4-Q3	-6.5	7.10	-0.9	*ns*
10th vs. 8th by Q4-Q3	8.2	6.38	1.3	*ns*
12th vs. 10th by Q4-Q3	0.8	7.25	0.1	*ns*

### Quartiles

As would be expected, there was a significant main effect of Quartile associated with reading rate (*p* < .001). There were also significant main effects of Quartile associated with each of the eye movement measures; faster reading rate quartiles were associated with fewer fixations per word (*p* < .001), fewer regressions per word (*p* < .001), and shorter fixation durations (*p* < .001). Main effects of Grade and Grade by Quartile interactions varied across measures and are described in the following sections.

### Silent Reading Rate

In all grade comparisons except between grades 6 and 8, the reading rates of older students were significantly faster than those of younger students (*p* < .001). There was also at least one significant grade-by-quartile interaction in each grade level comparison, except between grades 6 and 8). These interactions reveal the points at which reading rate increases in upper quartiles were greater than those occurring in lower quartiles. The first interaction involved a comparison of reading rate increases between grades 2 and 4 in the lowest two quartiles, and shows that these increases were 9.1 wpm larger in the second quartile compared to the lowest quartile (*p* < .001). Two additional interactions indicated that reading rate increases in the third quartile were larger than in the second quartile, these occurring between grades 4 and 6 (by 3.8 wpm, *p* = .039), and between grades 10 and 12 (by 9.0 wpm, *p* < .001). The fourth interaction indicated that reading rate increases between grades 8 and 10 in the highest quartile were significantly larger than those in the third quartile (by 13.4 wpm, *p* = .049). As a result of these grade by grade divergences in reading rate growth, the net difference in reading rate between grade 2 and grade 12 in the highest quartile was nearly double that seen in the lowest quartile (106 wpm versus 56 wpm).

### Fixations per Word

With two exceptions, students in upper grades made fewer fixations per word in comparison to those in lower grades (*p* < .001). The first exception was that students in grade 8 made more fixations per word than students in grade 6 (*p* = .001). The second exception was that the number of fixations per word did not change significantly between grades 10 and 12. There was only one significant grade-by-quartile interaction; the reduction in fixations per word between grade 2 and grade 4 was steeper in the second versus the third quartile (*p* = .029). Apart from this, reductions in fixations per word across grades did not differ significantly across adjacent reading rate quartiles. A strong negative correlation between fixations per word and reading rate was noted (*r* = -.80, *p* < .001).

### Regressions per Word

With two exceptions, students in upper grades made fewer regressions per word in comparison to those in lower grades (*p* < .001). The first exception was that students in grade 8 made more regressions per word than students in grade 6 (*p* = .003). The second was that the number of regressions per word did not change between grades 10 and 12. There was only one significant grade-by-quartile interaction, indicating that the reduction in regressions per word between grade 2 and grade 4 was steeper in the second versus the third quartile (*p* = .015). Apart from this, reductions in regressions per word across grades were not significantly different across adjacent reading rate quartiles. A moderate negative correlation between regressions per word and reading rate was noted (*r* = -.60, *p* < .001). In addition to the word-based regression rates, the overall proportion of regressive saccades was calculated. In the highest reading rate quartile, the proportion of regressions was 13.8% in grade 2 and 10.2% in grade 12 (a 26% difference). In the lowest quartile, the proportion of regressions was higher, with 19.7% in grade 2 and 18.8% in grade 12; a small difference of just ~4% across grades.

### Fixation Duration

Fixation durations declined significantly across all adjacent grade comparisons up through grade 10 (Table 6). There were no significant grade-by-quartile interactions. Notably, differences in fixation durations between grades 2 and 12 in the lowest quartile (the least efficient readers) were more than three times as large as those measured across these grades in the highest quartile (86 ms versus 27 ms; see Table 2). A moderate negative correlation between fixation duration and reading rate was noted (*r* = -.57, *p* < .001).

## Discussion

This research provides a description of eye movement behavior during authentic, productive silent reading across a large sample of typically developing elementary through high school students exhibiting different levels of silent reading efficiency. Across all levels of efficiency, the largest grade-to-grade changes in reading-related eye movements were seen in the elementary school grades. The trajectory of grade-to-grade changes in most eye movement measures appeared to level off in middle school. In high school, additional changes in reading-related eye movement measures tended to be modest and indices of increased reading efficiency were only seen in the upper quartiles.

Broadly speaking, reading rates can be fairly well approximated by multiplying the number of fixations (including those that follow both progressive and regressive saccades) by the average fixation duration (including saccade time), and converting this value to words per minute. For this reason, it is of interest that there were notable differences in the developmental trajectories of these two measures across reading rate quartiles. *In the upper (most efficient)* quartile, the overall pattern included reductions in fixations per word and corresponding increases in reading rate that continued through high school; yet declines in fixation duration tapered off after middle school. In the *lower* quartiles, reductions in fixations per word tapered off after elementary school, while declines in fixation duration continued through high school. As such, it seems that in high school, the small reading rate increments seen in the lower quartiles were largely a consequence of continuing declines in fixation duration. These results are discussed more fully in the following sections.

### Patterns of Development

As would be expected, reading rates were faster in the upper grades. Of more interest, however, was the observation that, in nearly every comparison between adjacent grade levels, reading rate increases were larger in the upper as opposed to the lower quartiles. The cumulative effect of this divergence becomes apparent when comparing absolute differences in reading rate across quartiles in the youngest vs. the oldest readers in our sample. While reading rates in the lowest quartile averaged 72 wpm in grade 2 and were only 56 wpm faster in grade 12 (128 wpm), reading rates in the highest quartile averaged 169 wpm in grade 2 and were 106 wpm faster in grade 12 (275 wpm)^1^. Taken together, these differences in reading rate increases between grades led to an ever-widening gap between the less and more efficient readers in a manner consistent with the “Matthew effect” [ 37 ].

While absolute differences in reading rate between grades 2 and 12 were largest in the most efficient readers, absolute differences in fixations, regressions, and fixation duration were larger in the *least* efficient readers. This potentially confusing circumstance is explained by the much higher initial (grade 2) fixation and regression counts and longer fixation durations in the less efficient readers. Calculated as a *percentage*, the differences in fixations and regressions per word between grade 2 and grade 12 were actually *smaller* in the less efficient readers. The percent difference in fixation durations, on the other hand, was larger in this group. Considered together, these differences suggest that reductions in fixations per word make a larger contribution to efficiency gains in the upper quartiles, while reductions in fixation duration do so in the lower quartiles. It would be of interest to examine this possibility more closely using a more sophisticated eye-tracking system.

*The Middle School Plateau.* Overall, reading rate increases were fairly smooth from grade to grade within each quartile. The exception to this pattern was the relative absence of reading rate increases in all quartiles when comparing grade 6 to grade 8; a plateau that was accompanied by an increase in fixations and regressions. Fixation duration, however, continued to decline between these grades. Several possible explanations for this discontinuity were considered. Systematic differences in the student sample seemed unlikely since the demographic characteristics of the sample were comparable across grades [see 6]. Features of the stimulus materials are more difficult to rule out as a contributing factor. As shown in Table 1, the Lexile scores of the passages increased fairly smoothly from grade to grade, as did the mean word length. The mean word frequencies across grades, however, were less consistent. The mean of the log word frequencies (MLWF) associated with the Lexile scores of the passages [see 38] declined most steeply between grades 4 to 6 and 6 to 8, after which they actually increased. The same pattern was seen using SUBTL word frequency norms for each passage based on the SUBTLEXUS corpus [32]. The SUBTL norms based on *unique* words declined most steeply between grades 2 to 4, remained steady to grade 6, and then declined again between grades 6 to 8. These variations in the progression of word frequency changes are notable but seem inadequate to fully account for a middle school hiatus in reading efficiency development; if word frequency effects on other measures of reading efficiency were considerable, for example, then an effect on fixation duration would be expected as well (e.g., [ 7, 19 ]), yet no such effect was apparent. Clearly, additional research will be required to gain a fuller understanding of the role of text complexity as well as other factors in modulating middle school reading efficiency development.

Notable in this connection is evidence that challenges associated with simply transitioning from elementary to middle school can contribute to stagnating growth in reading proficiency between grades 6 and 8. Research has documented declines in student achievement that coincide with this transition and there is evidence that such declines include significant drops in reading achievement per se that can persist through grade 8 or even longer [ 39, 40, 41, 42 ].

*High School Divergence.* Another notable finding in the quartile analysis was the continuation of reading efficiency increases across grades in the upper quartiles during high school, and a relative absence of reading efficiency increases in the lowest quartiles during these years. Between grades 8 and 10, growth in the lower three quartiles was barely half of that seen in the highest quartile, and between grades 10 and 12, there was essentially no reading efficiency growth at all in the lowest two quartiles; reading rates were stagnant and there was a trend toward making more fixations and regressions per word.

The number of fixations and regressions is known to increase when a reader encounters words that are difficult to comprehend or reading material becomes more challenging [ 43, 44, 45, 46 ]. In the present study, high school students in the highest reading rate quartile were notable in that they were the only students who achieved an average of one fixation per word or less. Those in the lowest two reading rate quartiles were averaging between 1.4 and 1.7 fixations per word. These higher fixation rates in the lower quartiles suggest that these students found the text to be more challenging; i.e., whether by necessity or habit, they had to make more fixations per word to decode grade-level text. The regression data are consistent with this view as well: In grade 12, students in the lowest quartile averaged more than three times as many regressions per 100-word test passage as compared to students in the highest quartile (34 versus 10 regressions). They also had a significantly higher *proportion* of regressive saccades as compared to students in the highest efficiency quartile (18.8% versus 10.2%).

Skilled readers who have, through reading practice, built up a large collection of sight words will identify many words in a single fixation, and sometimes even skip words that are highly predictable from the context [ 9, 47, 48, 49 ]. At the same time, developing and less-efficient readers who may be less familiar with many of the words they encounter are more likely to need multiple fixations to identify a word (e.g., while using sub-lexical analysis to construct a phonological representation, or mentally “sound out” the word). The cognitive effort associated with identifying unfamiliar words diverts attention that might otherwise be available for cognitive priming [ 50] and for the preprocessing of information in the parafoveal region [ 1, 4, 10, 22 ] thereby postponing the first steps in identifying subsequent words and further slowing the reading process. At a more global level, this less efficient reading behavior is more taxing on attention, comprehension, and memory; perhaps to the point that information is lost before the end of a sentence has been reached and connected meaning has been constructed [ 51, 52, 53, 54, 55]. Consistent with this view is research documenting an association between reading rate and comprehension [ 56, 57, 58, 59, 60, 61 ]. Considering the present results from this perspective, the slower reading rates and higher fixation and regression rates measured in high school students in the lower quartiles may suggest that many of these students have not developed their word recognition skills to the point that they can efficiently read and construct meaning from grade level material. Given that reading volume is a critical factor in becoming a better reader [ 37, 62, 63 ], these results might also suggest that students in the lower quartiles are simply not reading enough to improve their reading skills.

*The Development of Fixation Duration.* In comparison to the other eye movement measures described here, fixation duration showed a somewhat different pattern of development across grades. First, moving from the lower to upper grades there appeared to be a fairly smooth decline in fixation durations, with all quartiles converging toward mean durations in the range of 240-280 ms (this value includes the ~20-40 ms saccade time), with no irregularities in the middle school grades as there were in each of the other measures. Second, the decline in fixation durations across grades was steepest in the *lowest* reading rate quartile, with a decline of 86 ms between grades 2 and 12, as compared to a decline of just 27 ms across the same grade span in the *highest* quartile. Third, fixation durations in the highest, most efficient quartile did not decline at all after grade 8, at which point (after subtracting saccade time) they were comparable to those of skilled adult readers [ 19, 64 ].

Changes in fixation duration in the high school grades also appeared to be largely unrelated to changes in reading rate. Fixation durations continued to decline, for example, in the lower quartiles at the same time that these students were showing little or no growth in other measures of reading efficiency development. Indeed, in the lowest two quartiles there was a trend toward more fixations and regressions per word between grades 10 and 12 that was sufficient to offset much of the reading rate improvement that might otherwise have resulted from continuing declines in fixation duration. At the same time, fixation durations were no longer declining in the highest quartile, having already declined by grade 8 to what some research has suggested is the minimum amount of time required for lexical processing and associated oculomotor events (e.g., see [ 65 ], Fig. 1). Yet students in this quartile continued to increase their reading rates; an increase that could only have been achieved by making fewer fixations per word.

Taken together, one interpretation of the apparent disassociations between fixation duration and the other reading efficiency measures is that declines in fixation duration over grades might at least in part reflect maturational processes rather than increases in reading skill. This is not to suggest that reading ability and text difficulty do not also play a role; in both children and adults there is good evidence for word frequency, familiarity, and predictability effects on fixation duration [ 8, 19, 66, 67 ], and notably, these effects more pronounced in children as compared to adults [ 7, 68 ]. All in all, it seems likely that both maturational factors and reading experience contribute to age-related declines in fixation duration during reading. Perhaps most students follow a similar maturational time course, for example, but with text complexity effects superimposed on this baseline.

### Limitations

Despite the advantages of the simple eye movement recording device used in this research, it does not offer the resolution that might otherwise provide for additional insights into certain underlying processes during reading. Regressions, for example, can be divided into inter- and intra-word regressions. Intra-word regressions are more indicative of word level difficulties such as problems with lexical processing or oculomotor positioning errors, and account for 97% of regressions in fluent adult readers [69]. Inter-word regressions typically indicate comprehension-related processes at the sentence level, such as difficulties with semantics or syntax [ 68, 70, 71, 72 ]. The regression counts described in this report are short-range regressions (up to about three words in length); more refined distinctions within this range cannot be made using this device.

Additional limitations are associated with the reported estimates of fixation duration. The Visagraph does not segregate saccade time, and at the single word level does not divide fixation durations into first fixation, gaze duration, and total word reading time; measures that would enable more comprehensive analyses (c.f., [ 8, 20, 48 ]).

Another interesting point is related to reading mode. In the current study, children were asked to read silently. Contrary to initial concerns, even the youngest children (2nd grade) were able to do this without much difficulty. Although not focus of the present study, it would certainly be interesting to investigate the possibility of differential effects of reading mode on readers with varying reading skills and ages in future studies. This is especially so since previous studies with adults [ 11 ], adolescents [ 12 ], and children [ 8 ], point to specific differences in eye movement parameters during oral versus silent reading.

With regard to the study design, practical considerations dictated that a cross-sectional analysis be used rather than a longitudinal approach; a choice that is associated with some limitations. Systematic differences across the students in each grade group, for example, could have contributed to the pattern of results obtained. Based on the available demographic data, there is no indication that this occurred, yet the possibility cannot be ruled out. Relatedly, independent measures of reading ability were obtained from most participating schools, but differences in the assessment instruments and procedures used in each state limited the opportunity to make meaningful comparisons. As such, confidence in the present results, and in particular, the grade to grade developmental trajectories, would benefit from corroborating evidence obtained using a longitudinal design.

A difficult choice in cross-sectional research is whether to use one set of standardized passages for all grades, or different grade-leveled passages for each grade. If a single set of passages is used, the results obtained will likely reflect the probability that the passages are more difficult for younger students and easier for older students. On the other hand, confidence in the results obtained using different sets of grade-leveled passages depends on the reliability and validity of readability metrics. Despite this limitation, it was decided to use grade-leveled passages in this research due to the range of grades involved. The readability metrics associated with these passages suggested that they did provide a fairly uniform progression of grade-appropriate difficulty. It remains possible, however, that some variations in the reading efficiency development trajectory could have been due to qualitative variations in the test passages that were not detected using Lexile and word frequency measures, nor by the readability formulas used during the development and testing of the passages. Mitigating this possibility is the fact that the same grade leveled passages had been used in previous research [ 9 ] and yielded results that held up well in comparison with later research [ 73, 74 ].

### Conclusions

Cultivating the development of literacy is a fundamental goal of children’s formal education. Beginning in the early primary grades, children in countries with alphabetic writing systems learn their letters and the associated sounds, receive explicit instruction to increase their phonemic and graphemic awareness, are encouraged to read to increase fluency, and are taught vocabulary and cognitive strategies designed to increase comprehension (e.g., [53]). Yet national data on silent reading efficiency [ 6 ] indicate that half of all students in the U.S. complete high school with reading rates that are far below or at best comparable to typical conversational speaking rates in English. When reading is this slow and arduous, it is likely to be difficult for the reader to sustain the level of attention that close reading requires. Moreover, students who read this slowly are likely to be devoting a considerable portion of their cognitive resources to decoding and sounding out words or trying to figure out what words mean, and will therefore find it difficult to focus on the broader meaning of what they are reading. As in the old adage, it can be a matter of “not seeing the forest for the trees.” That many students find themselves in this situation is suggested by the results of the recent National Assessment of Educational Progress [ 75 ]. According to those results, nearly two-thirds (63%) of U.S. 12th grade students are not proficient in reading and 28% fail to demonstrate even a basic level of reading achievement.

The present results shed light on some of the underlying difficulties that less efficient readers are facing. In the lower two quartiles for reading rate, for example, the numbers of fixations and regressions per word in grade 12 were essentially the same as those seen in grade 6. This suggests that, like their younger counterparts, older students with below average reading rates are continuing to struggle with word identification and rely on sub-lexical processing strategies. While accumulating reading experience would be expected to improve word recognition and reduce fixations and regressions per word, the data suggest that students in the lower quartiles may not be accruing sufficient experience to offset the demands of increasing text complexity as they advance through school. To the extent that this is the case, it would seem crucial for these students to more fully develop their decoding skills and reading efficiency using appropriately leveled practice texts before advancing to more challenging material.

### Ethics and Conflict of Interest

The authors affirm that this research complies with the ethical standards outlined on the website of the Journal of Eye Movement Research. The authors note that Reading Plus/Taylor Associates Communications developed the Visagraph device used in this research. There are no conflicts of interest related to the publication of this paper.

### Acknowledgements

This research was supported by Reading Plus/Taylor Associates Communications, Winooski, Vermont, USA. Portions of these data were presented at the 18th European Conference on Eye Movements [ 76 ].
